# Human Wharton’s jelly mesenchymal stem cells-derived secretome could inhibit breast cancer growth *in vitro* and *in vivo*

**DOI:** 10.22038/ijbms.2020.42477.10020

**Published:** 2020-07

**Authors:** Mansoureh Mirabdollahi, Hojjat Sadeghi-aliabadi, Shaghayegh Haghjooy Javanmard

**Affiliations:** 1 Applied Physiology Research Center, Cardiovascular Research Institute, Department of Physiology, School of Medicine, Isfahan University of Medical Sciences, Isfahan, Iran; 2 Medicinal Chemistry Department, School of Pharmacy, Isfahan University of Medical Sciences, Isfahan, Iran

**Keywords:** Breast cancer, Growth inhibition, hWJMSCs, In vitro and in vivo, Secretome

## Abstract

**Objective(s):**

Controversial results have been reported regarding the anti-tumor properties of extracellular vesicles derived from mesenchymal stem cells (MSCs). The present study was conducted to evaluate whether secretome derived from Human Wharton’s jelly mesenchymal stem cells (hWJMSCs) may stimulate or inhibit breast cancer growth *in vitro* and *in vivo*.

**Materials and Methods:**

MTT assays was performed to determine anti-tumor effects of hWJMSCs-secretome on both MCF-7 and 4T1 tumor cells* in vitro*. Afterward, 4T1 breast tumors were established in different groups of Balb/C mice (12 mice/group). The tumor sizes were monitored in different treatment groups and at day 30 post-tumor inoculation (PTI), blood samples were obtained and 6 mice of each group were sacrificed for hematological and histopathological assays. The rest of the mice in each group (n=6) were left alive up to day 120 PTI to determine survival rate.

**Results:**

We found that hWJMSCs-secretome can inhibit growth of MCF-7 and 4T1 tumor cell lines in vitro. Moreover, intratumoral administration of hWJMSCs-secretome resulted in signiﬁcant tumor growth inhibition and improvement of hematological indices in vivo and prolonged survival rate of tumor bearing mice.

**Conclusion:**

According to our findings, hWJMSCs-secretome could be considered a potent anti-tumor agent, however, further investigation should be done on other cancer models.

## Introduction

It has been shown that different types of mesenchymal stem cells (MSCs) have tropism for the tumor microenvironment (1). Considering that, several studies prompted to investigate the potential tumorigenic or anti-tumor effects of MSCs. In this regard, some studies showed that MSCs could enhance tumor growth *in vivo *(2), while the others revealed that MSCs can exert anti-tumor effects (3-5).

It has been shown that tail vein injection of bone marrow MSCs (BMMSCs) inhibited the *in vivo* established Kaposi’s sarcoma (6). Moreover, in another study the intraperitoneal (IP) injections of BMMSCs exerted inhibitory effects against non-Hodgkin’s lymphoma generated by IP injections in severe combined immunodeficient (SCID) mice (7). Furthermore, the effect of human umbilical cord mesenchymal stem cells (hUCMSCs) on metastatic breast cancer cells (MDA-MB-231) was examined previously (8). The findings of this study showed that, intravenous injections of hUCMSCs significantly attenuated MDA-MB-231 tumor growth compared with the control group (8). 

Regardless of the above mentioned studies and similar findings by other studies, the exact tumorigenic or anti-tumor effects of MSCs is still a matter of debate (9, 10). To figure this out, many studies have been conducted to characterize the mechanisms of action by which MSCs can modulate tumor growth (9). More recently, researchers have focused on extracellular vesicles (EV) released by MSCs in tumor microenvironment. Several studies highlighted the pivotal role of EVs in cell-cell communications as well as in the development of various malignancies (9). It has been demonstrated that these EVs can act paracrinally and influence the physiological functions of other cells in different ways including direct contact with target cells, delivering microRNAs or proteins to target cells, which leads to induction of different epigenetic changes (11-14).

According to previously published papers, EVs derived from MSCs showed different anti-tumor and tumorigenic effects (15-17). Recently, the inhibitory or stimulatory effect of microvesicles (MVs) derived from human BMMSCs was evaluated on HepG2 hepatoma, Kaposi’s sarcoma, and SK-OV-3 ovarian tumor cell lines *in vitro* and *in vivo *(17). According to the results of this study, intratumoral administration of MSC-MVs significantly inhibited growth of the above-mentioned tumors (17). Also, another study showed that MVs derived from human embryonic MSCs can inhibit proliferation of leukemia cells in a concentration-dependent manner (18). In addition, it was found that MVs derived from human umbilical cord Wharton’s jelly mesenchymal stem cells (hWJMSCs) can significantly attenuate the bladder tumor T24 cell growth both *in vitro* and *in vivo *(19). Altogether, there are several studies suggesting that the tumorigenic or anti-tumor effects of EVs can be dependent on the MSC sources from which EVs are derived as well as different types of cancers (20-22). In the present study, we aimed to evaluate whether EVs derived from human Wharton’s jelly MSCs may stimulate or inhibit experimental breast cancer growth. To this end, we have evaluated: (a) the effects of hWJMSCs-derived secretome on proliferation of 4T1 and MCF-7 tumor cells *in vitro*; (b) the *in vivo* effect of intratumoral administration of secretome in tumor models generated by subcutaneous injection of the 4T1 cell line in Balb/C mice. 

## Materials and Methods

***Cell cultures***

Human breast cancer cell line (MCF-7) and mouse mammary tumor cell line (4T1) were used throughout this study. The cell lines were obtained from Iranian Cell Bank of Pasteur Institute. Cells were cultured in T75 tissue culture flasks (Becton Dickinson, USA) containing RPMI-1640 (Gibco) medium supplemented with 100 U/ml penicillin, 100 µg/ml streptomycin (Gibco, Grand Island, NY) and 10% FBS. The cultures were incubated in a humidified atmosphere at 37 ^°^C containing 5% CO_2_.

***Source of MSCs***

MSCs were isolated from Wharton’s Jelly (umbilical cord, UC) of three healthy subjects (19-35 years old) who underwent cesarean-section. The hWJMSCs were isolated using a modified version of procedure introduced by Lu *et al. *(23). Briefly, the umbilical cord tissue was washed with PBS solution to remove red cells and arteries, and then cut into approximately 0.5–1 cm pieces. The obtained pieces were transferred to a sterile Petri dish containing low glucose Dulbecco’s Modified Eagle’s medium (DMEM; Gibco, Grand Island, NY) supplemented with 10% fetal bovine serum (FBS, Gibco) and incubated at 37 ^°^C in a humidified 5% CO_2_ atmosphere. Cultured tissues were incubated for a period of two weeks to allow MSCs migrate out of the umbilical cord tissue and adhere to the Petri dish. In this period, the medium was changed every three days to remove dead cells and debris. Afterward, the adhered MSCs were harvested by 0.25% trypsin and subcultured in tissue flasks for further experiments. 

***Characterization of MSCs***

The hWJ-MSCs were examined using an optical microscope and evaluated for the presence of CD14, CD34, CD45, CD73, CD90, and CD105 markers using flow-cytometry. Furthermore, the differentiation capability of these MSCs to different lineages was investigated. For flowcytometric analysis, cells were washed twice with PBS to remove cell debris and then detached using trypsin 0.25%. Cells were stained for 25 min at 4 ^°^C using fluorescence-labeled antibodies. Also mouse IgG antibodies conjugated with identical concentrations of FITC and PE were used as negative control. Each flow-cytometry experiment was performed with at least 10000 events using an FACS Caliber^®^ flow-cytometer (BD biosciences, USA).

***Isolation of hWJMSC-Secretome***

To isolate the secretome, hWJMSCs cultures at 4th passage with a confluency of 80–90% were chosen. At this time, the supernatant was removed and cells washed two times with phosphate buffer solution (PBS), and then, 10 ml of serum free DMEM medium was added to each flask and incubated for 48 hr. After incubation, the supernatants were collected and centrifuged at 2000 g for 10 min to remove cell debris and dead cells. Then, the collected supernatant was dried using a freeze-dryer device (Freezdryer, Christ. a2-4, Germany) and stored at -80 ^°^C before using. 

***MTT assays***

In order to evaluate the possible cytotoxic effects of the collected secretome derived from hWJMSCs, we examined the IC_50_ of this secretome in different concentrations (2, 4, 6, 8, 10, 15, and 20 mg/ml) using MTT (3-(4, 5-dimethyl-2-thiazolyl)-2, 5-diphenyl-2-Htetrazoliumbromide) assay. In summary, the MCF-7 and 4T1 cancer cells were plated at a density of 4×10^4^ cells/well and 3×10^4^ cells/well, respectively in a 96-well plate, containing low glucose DMEM for 24 hr. Then, the cells were treated with different concentrations of hWJMSCs-secretome in a total volume of 180 µl DMEM for 48 hr. afterward, 20 µl of MTT solution was added to the wells and incubated for another 4 hr. Once the reaction was terminated, the supernatants were discarded and 150 µl of dimethyl sulfoxide was added to dissolve the formazan crystals. Finally, the plates were analyzed at 490 nm wavelength by an ELISA reader (Biotek, USA).

***Study design, tumor transplantation, incidence, and latency***

**Figure 1:**
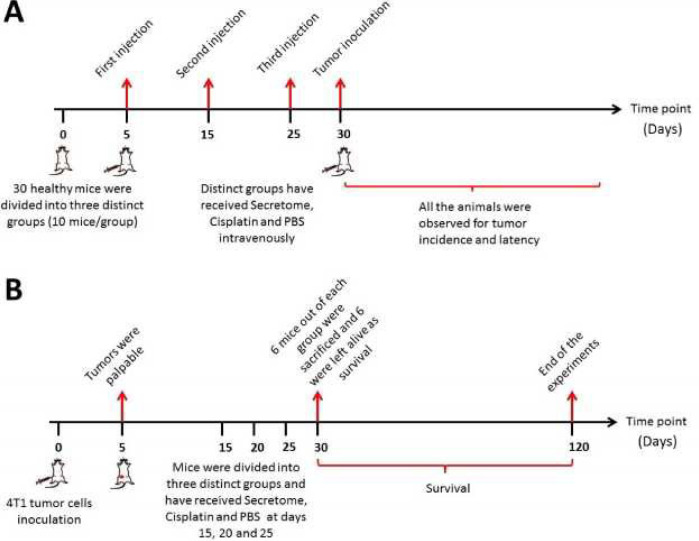
Schematic representation of the study design

The present study was conducted in two independent parts; (1) in the first part, thirty 4-6 weeks old female Balb/C mice (Royan Institute, Isfahan, Iran) were allowed to adopt to the new environment for 1 week and then divided into three groups (10 mice in each group) that received either secretome (20 mg of secretome per injection), cisplatin (0.2 ml per injection with the concentration of 10 mg/kg), and/or PBS (1 ml per each injection) as treatment, positive, and negative groups, respectively. Three intravenous injections were made for an interval of 10 days (at days 5, 15, and 15) and then, at day 30 the subjected groups were inoculated with 3.5 ×10^6^ 4T1 cells. Afterward, the subjects in all groups were examined each day to detect the appearance of tumor masses. Thus, the incidence (% of the tumor bearing mice in each group) and the latency (the days taken for the mice to develop first tumor mass) of the tumor masses were observed and recorded (Figure 1A). (2) In the second part of the present study, mice were inoculated with 4T1 tumor cells and once the tumor masses appeared and reached approximately 1.5 Cm^3^, then animals were divided into distinct groups and received different treatment regimens (Secretome, cisplatin, and PBS) in order to evaluate the antitumor effects of hWJMSCs-derived secretome *in vivo*. Schematic representation of the study design is presented in Figure 1B.

***Evaluation of the anti-tumor effects of hWJMSCs-secretome in vivo***

To evaluate the *in vivo* effects of hWJMSCs-secretome against murine breast carcinoma cells (4T1), 4–6 weeks old female Balb/C mice were used. In this regard, as mentioned previously 3.5 ×10^6^ 4T1 cells was inoculated subcutaneously into the syngeneic animals under sterile conditions. Then, mice were randomized into 3 groups: (1) tumor-bearing mice received injections of saline (positive control), (2) tumor-bearing mice received injections of hWJMSCs-secretome (three intratumoral injections at 5 days intervals and with 20 mg of secretome in a volume of 1 ml of PBS (vehicle) per injection), (3) tumor-bearing mice received injections of cisplatin (three intratumoral injections with 0.2 ml per injection and 10 mg/kg of cisplatin concentration). Moreover, two additional groups of healthy mice: (4) those receiving saline injections alone to serve as absolute negative controls, (5) and mice receiving 20 mg secretome were included in the present study (to evaluate hematological changes following administration of secretome). During the study, we monitored the animals for activity and physical conditions every day, and every 3 days the body weight and tumor mass of mice were measured. To determine tumor mass, growth of the implant was monitored by caliper measurements and calculated using the formula 1/2a ×b^2^, where a stands for the long diameter and b is the short diameter. Twelve mice were included in each group and observed for signs of morbidity during the experimental period. Six mice out of each group were sacriﬁced 30 days after tumor inoculation by cervical dislocation in accordance with the Animal Ethics Guidelines and the remaining 6 mice in each group were left alive to observe survival rate. Moreover, the tumor masses of different treated and untreated groups were immediately frozen in optimal cutting temperature (OCT) compound and sectioned (Tissue-Tek, Bayer AG, Switzerland) for further histological analysis. All animal experiments were performed under the Guidelines for the Care and Use of Laboratory Animals set by Isfahan University of Medical Sciences. Schematic representation  of the study design is presented in Figure 1B.

***Determination of the body weight***

The body weight of mice was measured every three days from the beginning of the treatment course until the end of study using a digital analytical balance. The percentage of the body weight changes was calculated at the end of the experimental period and compared with the initial body weight. To measure the actual body weight changes, the weights of tumors were subtracted. The formula used to calculate percentage weight gain is as follows (24):

Percentage of the body weight change (gain or loss)= [(final body weight-initial body weight) X 100]/initial body weight

Actual body weight=body weight at day 30 – weight of tumors

Percentage of the actual body weight change (actual body weight change %)=[(actual body weight–initial body weight) X 100]/initial body weight

***Histopathological examinations***

Subcutaneous tumors were recovered, ﬁxed in 10% formalin, and embedded in parafﬁn. Sections of 3 μm thickness were prepared, mounted on frosted-end glass slides, deparaffinized and stained with Hematoxylin and Eosin (H&E). The histologic examinations were done by light microscopy at 10× and 40× magnifications. Two expert pathologists, who were blinded to the treatment groups, examined the tissue sections. Finally, human cancer grading system (Bloom and Richardson technique) was used to grade the tumors (25).

***Hematological assays***

The blood samples were obtained from different treated and untreated groups (secretome treated tumor bearing mice, cisplatin treated tumor bearing mice, PBS treated tumor bearing mice, secretome treated non-tumor mice, and healthy controls) and collected in tubes pre-treated with EDTA. Afterward, an automated cell count analyzer (Sysmex KX-21, Japan) was used to measure the full blood count (FBC). Several parameters of hemoglobin concentration (HGB), red blood cell (RBC), red blood cells distribution width (RDW), mean cell volume (MCV), mean corpuscular hemoglobin (MCH), mean corpuscular hemoglobin concentration (MCHC), platelet, white blood cell count (WBC), neutrophils, lymphocytes, monocytes, eosinophils, and basophils were determined for each sample. To this end, 20 μl of the whole blood was aspirated by a sampling probe and the result of analysis was obtained accordingly. Furthermore, manual smears of blood samples were examined by a single expert hematologist to confirm the result obtained by automated cell counter. 

***Statistical analysis***

All data were expressed as mean ± standard deviation (SD). The normal distribution of data was tested using the Kolmogorov-Smirnov tests. The median values between groups were compared by Kruskal-Wallis H test and the differences between the two groups were analyzed by the Mann–Whitney U test followed by Bonferroni’s correction. The effects of hWJMSCs-secretome on mice body weight gain was analyzed by analysis of variance (ANOVA). Kaplan-Meier survival curves were analyzed using log-rank tests. *P<0.*05 was considered as statistically significant. All analysis was performed using the GraphPad Prism software version 5 (San Diego, California, USA).

## Results

***Characterization of MSCs***

The data on morphological, flowcytometric, and differentiation potential of hWJMSCs to different lineages is available in the other works published by the same group elsewhere (26-28).

***hWJMSCs-derived secretome inhibits tumor growth in vitro***

**Figure 2:**
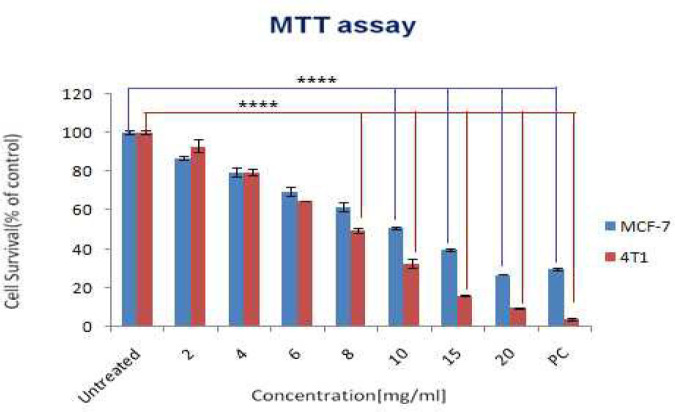
The anti-tumor effects of Human Wharton’s jelly mesenchymal stem cells derived secretome on MCF-7 and 4T1 tumor cells were determined by MTT assay. Different concentrations of secretome were considered. Statistical differences were calculated between untreated controls and cells treated with hWJMSC-secretome (all experiments were performed triplicated (n=3)). PC refers to positive control (10 mg of cisplatin). (* *P<*0.05, ** *P<*0.01, *** *P<*0.001, and **** *P<*0.0001)

The cytotoxic effects of the hWJMSCs derived secretome were evaluated at different concentrations on both MCF-7 and 4T1 tumor cells. The results of three replicates showed that the hWJMSCs-secretome could inhibit MCF-7 and 4T1 cells dose-dependently (Figure 2). The IC_50_ was determined as ≥10 mg/ml (Figure 2).

***hWJMSCs-derived secretome therapy decreased tumor incidence, size, and weight and increased tumor latency***

**Figure 3:**
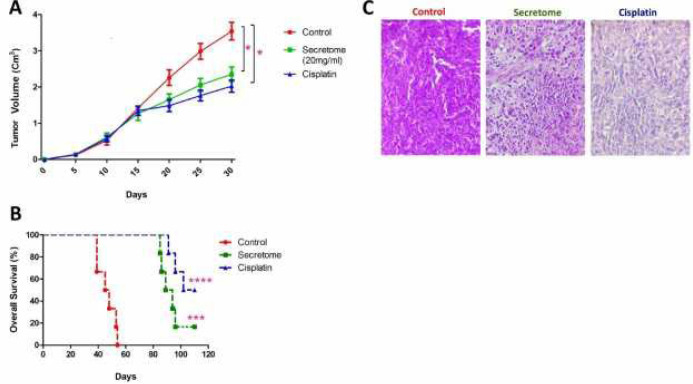
The effects of hWJMSCs derived secretome on tumor size progression and survival rate of mice bearing breast cancers

Palpable tumors were evident in all mice subcutaneously inoculated with 4T1 tumor cells. The subjects in the treatment groups (Groups 2 and 3) showed a significantly higher latency period and lower tumor incidence (*P<0.*05) compared with the control tumor bearing group (Group 1). Moreover, no statistical differences were found among treatment groups (Groups 2 and 3). According to the results, either 20 mg/ml of hWJMSC derived secretome or the cisplatin (0.2 ml per injection with a concentration of 10 mg/kg) significantly reduced tumor size and weight (*P<0.*05) compared with untreated negative control (PBS). The details of the findings are presented in Table 1 and Figure 3A.

***The hWJMSCs derived secretome inhibits tumor progression and prolonged survival rate in tumor bearing mice***

The tumor sizes were measured daily during the experimental period (30 days). The mice in both treatment groups (Groups 2 and 3) exhibited a slower size growth and smaller median tumor size (<1.98 cm^3^) compared with the control group (Group 1, PBS treated). However, the mice of the negative control group showed a rapid tumor size growth in a shorter time with the median size of 3.46 cm^3^. The differences of tumor progression between both treatment groups and untreated tumor bearing mice were significant (*P<0.*05). Moreover, treatment of tumor bearing mice increased their survival rate significantly (*P<0.*001) compared with the untreated mice (PBS). Also, no statistical differences were observed between the treatment groups (i.e., those receiving secretome or cisplatin). Furthermore, at the end of the study a subject from the secretome treated group and 3 subjects from cisplatin treated group were still alive. The results are illustrated in Figures 3 A and B.

***Body weight measurement***

The mice in all groups (non-tumor mice (PBS), non-tumor mice (secretome treated), tumor bearing mice treated with hUCMSC-Secretome, and tumor bearing mice treated with cisplatin) except the PBS-treated tumor bearing group gained weight gradually throughout the experimental period. The data of median body weight for mice of different groups is presented in Table 2. The differences of median body weight were not significant at day 0 of the experimental period between the treatment groups compared with the negative control (*P*=0.985). However, at the end of the experimental period, significant statistical differences were observed in terms of median body weight between treatment groups (secretome treated non-tumor and tumor bearing groups or cisplatin treated group) and the untreated tumor bearing control group (*P*=0.046). Moreover, the body weight of the secretome treated tumor bearing group was slightly higher than the other three groups. Furthermore, the overall change in body weight (percentage of body weight change) between all of the four above-mentioned groups was statistically significant (*P*=0.000). Also, the actual body weight change between treatment groups and untreated tumor bearing mice was found to be significant (*P*=0.000).The findings of the body weight in different treatments as well as untreated tumor bearing and non-tumor groups are presented in Table 2. The weight loss is presented by negative values.

***Macroscopic and microscopic evaluation of tumors***

The tumor masses in both treatment groups and PBS-treated tumor bearing group were examined both macroscopically and microscopically. The initial macroscopic findings showed that the tumors of control group were larger, solid, and dense with necrotic and hemorrhage morphology. However, the tumors in treatment groups (secretome or cisplatin) were found to be softer, less hard, as well as smaller in size. We observed that the tumor masses were almost shrunk in both of the treatment groups during the *in vivo* experiments. The histological assessments exhibited plump tumors in the negative control group compared with the hWJMSCs-secretome or cisplatin treated groups. However, the histopathological assessments showed that the tumors in untreated mice and mice treated with hWJMSC-secretome or cisplatin had the same histological grading (grade III) (Figure 3C). 

***Hematological parameters***

The hematological values of the healthy non-tumor mice were taken as the reference values. According to the findings, the treatment groups (secretome treated non-tumor or tumor bearing mice and cisplatin treated tumor bearing mice) had hematological values higher or closer to the non-tumor mice. However, the untreated tumor bearing mice (PBS-group) had lower values in terms of hematological values. The mean±SD of hematological values were presented in Table 3. The hWJMSCs-secretome or cisplatin treated groups showed a higher level of total white blood cell (TWBC), red blood cell distribution width (RDW), polymorphs, and monocytes compared with either non-tumor or untreated tumor-bearing groups. The differences were only significant between treatment groups (above mentioned three treated groups) and untreated tumor bearing group. Moreover, the differences were not significant between the hWJMSCs treated group and the mice treated with cisplatin (Table 3). 

A) Three groups of mice (10 mice/group) received hWJMSC-derived secretome, Cisplatin, and PBS and surveyed for tumor incidence and latency (preventive effects). B) The 4T1 cells were inoculated into three different groups of mice (12 mice/group). At days 15, 20, and 25 distinct groups received hWJMSC-derived secretome, Cisplatin, and PBS. Then 6 out of 12 mice were sacrificed in each group and the remaining 6 mice observed for survival

hWJMSC: Human wharton’s jelly mesenchymal stem cells; PBS: Phosphate-buffered saline

hWJMSC: Human wharton’s jelly mesenchymal stem cells

**Table 1:** The tumor properties of secretome treated mice compared with the untreated tumor bearing controls (PBS group)

Tumor		Groups		
	PBS-treated	Cisplatin-treated	Secretome-treated (20 mg/ml)	*P-value*
**Incidence (%)**	90	80	80	0.478
**Latency (days)**	6.6±1.3	16.2±2.5	18.9±2.2	0.012
**Size ** **(cm** ^3^ **)**	3.48±0.6	1.82±0.45	2.18±0.5	0.015
**Weight (g)**	3.22±0.6	1.42±0.25	1.96±0.43	0.005

Data are expressed as the Mean±SD; PBS: Phosphate-buffered saline

A) The size of tumors in both treatment mice groups and untreated tumor bearing mice were measured and compared. B) Kaplan-Meier survival curves were analyzed using log-rank tests and the statistical differences between treated groups and the untreated tumor bearing group were calculated. C) Representative graphs from tumor tissues stained with Hematoxylin and Eosin (H&E). Legends: control; untreated tumor bearing mice (PBS), secretome; hWJMSCs-secretome treated mice, Cisplatin; Cisplatin treated mice. (* *P<*0.05, ** *P<*0.01, *** *P<*0.001, and **** *P<*0.0001)

hWMSC: Human wharton’s jelly mesenchymal stem cells; PBS: Phosphate-buffered saline

**Table 2:** Body weight of mice treated with human Wharton’s jelly mesenchymal stem cells-secretome and Cisplatin compared with the controls

Body weight (g)	Groups					P-value*
	Non-tumor (secretome)	Non- tumoric (healthy)	PBS-treated (tumor bearing)	Cisplatin-treated	Secretome-treated	
**Body weight at day 0 **	17.9±1	17.2±1.5	18.1±0.9	18.6±1.1	18.5±0.9	0.985
**body weight at day 30 **	23.2±1.4	22±1.3	16.4±1	23.5±0.8	24.1±1.3	0.046
**Body weight change (%)**	29.6	27.9	-9.3	26.3	30.27	0.000
**Actual body weight change (%)**	29.6	27.9	-25.58	18.7	19.67	0.000

Data are expressed as the Mean±SD. * Kruskal-Wallis test was conducted and values are expressed statistically significant at *P≤*0.05

**Table 3:** The hematological effects of the human Wharton’s jelly mesenchymal stem cells -secretome on breast cancer bearing mice

Groups							
Parameters	Non-tumor (secretome)		Non-tumor (healthy)	PBS (tumor-bearing)	Cisplatin-treated	Secretome treated (20 mg/ml)	*P-value* ^*^
**WBC (10** ^3^ **/µl)**	4.5 ± 0.5		4.2 ± 0.5	3.6 ± 0.4	4.2 ± 0.2	4.8 ± 0.5	0.143
**Lymphocytes (%)**		68.4 ± 3.1	66.4 ± 3.1	53 ± 12.4	65 ± 13.4	66 ± 10.4	0.012
**Neutrophils (%)**	30 ± 9.2		33 ± 9.2	45 ± 16.5	34 ± 10.5	33 ± 14.7	0.005
**Monocytes (%)**	1.8 ± 0.6		1.3 ± 0.6	1.8 ± 0.5	1.3 ± 0.6	1.2 ± 0.6	0.675
**Eosinophils (%)**	0.4 ± 0.4		0.6 ± 0.4	0.8 ± 0.6	0.5 ± 0.5	0.6 ± 0.5	0.568
**Basophils (%)**	0.1 ± 0.1		0.3 ± 0.5	0.5 ± 0.4	0.4 ± 0.3	0.3 ± 0.3	0.812
**RBC (10** ^6^ **/µl)**	7.9 ± 0.9		8.1 ± 0.9	7.8 ± 0.6	8 ± 0.4	7.6 ± 1.0	0.124
**HGB** ** (g/dl)**	13.9 ± 1.0		13.2 ± 1.0	12 ± 0.5	12.6 ± 0.8	13.1 ± 0.6	0.118
**HCT (%)**	44 ± 4.0		45 ± 4.0	41.8 ± 6.2	48.3 ± 5.75	44.7 ± 4.2	0.954
**MCV (fl)**	66.1 ± 2.0		64.2 ± 1.5	66 ± 2.7	66.5 ± 6.75	66.5 ± 1.7	0.015
**MCH (pg)**	22.1 ± 0.8		20.4 ± 0.6	21.5 ± 1.0	21 ± 2.5	21.5 ± 2.1	0.975
**MCHC (g/L)**	34 ± 1.0		34.5 ± 1.2	36.8 ± 1.2	34.2 ± 1.7	35 ± 2.5	0.012
**RDW (%)**	14.1 ± 0.5		11.1 ± 0.5	13.5 ± 1.2	13.4 ± 1.7	12.9 ± 1.5	0.010
**Platelets (10** ^3^ **/µl)**	747 ± 135		849 ± 140	673 ± 192	735 ± 190	752 ± 130	0.048

Data are expressed as the Mean±SD. * Kruskal-Wallis test was performed and the differences compared with the untreated tumor bearing animals (PBS). Values are statistically significant at* P≤*0.05

WBC: White blood cell; RBC: Red blood cell; HGB: Hemoglobin; HCT: Hematocrit; MCV:Mean corpuscular volume; MCH: Mean corpuscular hemoglobin; MCHC: Mean corpuscular hemoglobin concentration; RDW: Red cell distribution width; PBS: Phosphate-buffered saline solution

## Discussion

The application of MSCs as potential anti-cancer agents has been investigated previously; however, controversial results were obtained in the preclinical studies (29, 30). It has been suggested that alternative measures such as exosomes, microvesicles, or secretome derived from different sources of MSCs can exert anti-tumor effects and potentially may be used as a supplement for the existing preventive and therapeutic modalities (31). Here, our study highlights the potential effectiveness of the hWJMSCs derived secretome against breast cancer.

According to our findings, the mice that received hWJMSCs derived secretome or cisplatin had higher tumor latency as well as lower tumor incidence compared with the untreated negative control (Table 1). Up to now, numerous attempts have been made to delay cancer onset both in healthy subjects or subjects who are at high risk of cancer development (32). Thus, our findings revealed that the breast cancer onset and incidence was reduced by hWJMSCs-secretome. In this regard, previous studies have also demonstrated that exosomes or microvesicles derived from MSCs potentially can inhibit mutagenic activity of the malignant cells (33, 34). Hence, we assumed that hWJMSCs-secretome may reduce tumor incidence and latency by inhibiting their mutagenic activity.

The effects of hWJMSCs-secretome on tumor size, weight, and progression was also examined. The result of the macroscopic evaluations demonstrated that treatment of tumor bearing mice with 20 mg/ml of hWJMSCs-secretome can slow down development of the breast tumors (tumor progression) with lesser tumor weight and lower tumor size compared with the PBS-treated controls (Table 1 and Figure 3A). Considering the reduced tumor size and weight in hWJMSCs-secretome treated group, it seems that this kind of treatment can reverse the tumorigenesis of the mice 4T1 breast cancer cells. The cancer development process can be divided into three main stages of initiation, promotion, and progression (35). It is assumed that preventive agents mainly act via intervening at the initiation or promotion stages of cancer development, while anti-tumor agents can inhibit tumor progression. Since the hWJMSC derived secretome decreased the tumor incidence (initiation of tumor development) and decreased tumor size and weight (tumor promotion and progression), we speculate that hWJMSCs-secretome can inhibit tumor growth probably by intervening at each of these three main stages. Moreover, our findings are consistent with other *in vivo *results that showed that hWJMSCs derived microvesicles may modulate tumor progression (19). Furthermore, the results of the present study indicate that the use of hWJMSCs-secretome can increase tumor latency and prolong the survival rate of tumor bearing mice. These results highlight the preventive and therapeutic anticancer effects of hWJMSC derived secretome.

Some of the breast lesions in our study were found to have almost disappeared at the end of the study. Since the chronic administration of low doses of chemotherapeutic drugs can eliminate or diminish tumors (36), it is quite possible that the secretome derived from hWJMSCs behaves similar to chemotherapeutic drugs or estrogen-lowering agents that are used in clinic at the time. Compared with chemotherapeutic agents, the use of hWJMSCs-derived secretome as an anticancer treatment has several advantages such as acting in a tumor specific manner, inducing or augmenting antitumor immune responses, and also inducing growth inhibition of different tumors (37). Although the MSCs derived exosomes or microvesicles may induce such antitumor effects, due to the MSCs-derived secretome containing different constituents (including cytokines, different interfering RNAs, etc.) it may inhibit tumor progression more effectively (37, 38). In the last decade, many studies attributed the anticancer effects of MSCs derived microvesicles to micro-RNAs (38). Thus, anti-tumoral effects of hWJMSCs-secretome may also be attributed to the micro-RNAs or other types of interfering RNAs, as well as cytokines and other antimitotic compounds. However, further investigations are needed to identify the agents which are responsible for these effects.

Since, different tumors can affect the health related indices, we also examined the body weights and hematological parameters of the animals in all groups. The blood parameters are investigated in cancer patients both before and after implementing treatment measures and it has been reported that poor blood parameters are associated with prognosis of malignancies (39). Moreover, in the case of patients with breast cancer, abnormal blood parameters have been observed to be associated with poor prognosis of cancer (39). Based on the results, the hWJMSCs-secretome or cisplatin treated groups had significantly higher body weight at the end of the study compared with the non-treated tumor bearing mice (Table 2). Moreover, the subjects in both of treatment groups had blood parameters closer to the healthy subjects (PBS-treated non-tumor mice). However, the untreated tumor bearing mice (PBS) had a lower level of lymphocytes RBC, HGB, HCT, and platelets compared with both of the treatment groups (Table 3). In this regard, cancer patients also have been reported with lower levels of RBC, HGB, MCV, MCH, MCHC, and lymphocytes and with higher levels of RDW, TWBC, and polymorphs either before or after treatment processes (39, 40). Higher body weight may indicate the good health condition of treated mice groups. Also, higher levels of lymphocytes can be associated with good prognosis and higher levels of platelets and HGB could be helpful in recovery from the injury due to cancer. In accordance, the results of the present study indicate that hWJMSCs-secretome can alter the hematological parameters to ameliorate the induced breast cancers. 

One of the important tools for the evaluation of the cancer prognosis is the histological grading of cancers (25, 41). In this regard, three criteria have been set up to score breast cancers based on cellular pleomorphism, mitotic activity, and tubular formation by cancer cells (41). Although our results revealed anti-tumor activity of hWJMSCs-secretome, according to our findings, the breast cancers that developed in the hWJMSCs-secretome or cisplatin treatment groups were mainly of grade III and had no difference with the untreated tumor bearing group (PBS group)(Figure 3C). It is probable that the doses of secretome or cisplatin used in the present study were not optimized, and higher doses of the subjected therapies would decrease tumor grade (down to grade I or II).

Altogether, we found that hWJMSCs-secretome can inhibit tumor growth and there may be different possible mechanisms by which hWJMSCs-secretome induces tumor growth inhibition.

## Conclusion

The administration of hWJMSCs derived secretome prior to cancer induction (preventive model) showed significant anti-cancer activity against breast cancer. We found that hWJMSCs-secretome could alleviate breast cancer progression via modulation of hematologic parameters. Moreover, slower tumor progression, lower tumor size and weight, longer latency period, and prolonged survival rate were observed in tumor bearing mice treated with hWJMSCs-secretome. Regarding to the obtained results, it can be concluded that hWJMSCs-secretome can be effective in cancer therapy; however, this claim needs further investigations in other cancer models.
